# Clinical Photography in Orthodontic Practice: Insights from a Nationwide Survey in Spain

**DOI:** 10.3390/jcm14061984

**Published:** 2025-03-14

**Authors:** Brezo Suárez-Solís, Carlota Suárez-Fernández, Juan Suárez-Solís, Alberto Badía, Maider Olabarria, Teresa Cobo

**Affiliations:** 1 Clínica Dental Suárez Solís, 33401 Avilés, Spain; 2 Department of Surgery and Medical-Surgical Specialties, School of Medicine and Health Sciences, University of Oviedo, 33003 Oviedo, Spain; suarezcarlota@uniovi.es (C.S.-F.); dra.cobo@iaodontologia.com (T.C.); 3 Orthokit App, C/de la Colina, 164, Torremolinos, 29620 Málaga, Spain; contacto@orthokit.es; 4 Instituto Asturiano de Odontología, C/Catedrático José Serrano, 10, Bajo, 33006 Oviedo, Spain; maiderolsoaz@gmail.com

**Keywords:** clinical photography, orthodontics, dental records, DSLR cameras, reflex cameras, surveys and questionnaires

## Abstract

**Background/Objectives:** Clinical photography is an essential component of orthodontic records, alongside radiographs, dental scanners, and cone beam computed tomography. However, neither the American Association of Orthodontists nor the Spanish Society of Orthodontics provides a standardized protocol for dental photography. This study aimed to evaluate the current practices, challenges, and training needs related to clinical photography among orthodontists in Spain. **Methods:** A cross-sectional survey was designed using Google Forms^®^ and distributed to orthodontists practising in Spain. A sample size of 303 participants was calculated (95% confidence level; 0.1 precision). The questionnaire covered photographic practices, equipment usage, self-assessed skills, interest in training, and legal considerations. Descriptive and inferential analyses were performed using R software (significance level 0.05; version 4.4.1). **Results:** A total of 304 valid responses were analyzed, with a predominance of female participants (77.96%) and a mean age of 37.54 years (SD: 9.08). Digital single-lens reflex (DSLR) cameras with macro lenses and ring flashes were the most used equipment (68.09%). The primary software for cropping and editing images were Windows Photos (28.95%) and MacOS Photos (16.12%). Male participants rated their photography skills higher than female participants (*p* = 0.003), and those with full-time orthodontic training considered their skills better than those with other types of training (*p* = 0.014). Photography was most valued for diagnosis in the exclusive orthodontics group (*p* = 0.019). Additionally, 75.99% of respondents expressed interest in improving their photography skills through specialized courses. **Conclusions:** This study highlights significant variability in photographic practices among orthodontists in Spain. The findings emphasize the need for standardized photography protocols to enhance diagnostic accuracy, clinical documentation, and professional training.

## 1. Introduction

Clinical photography has been essential to orthodontics, facilitating diagnosis, treatment planning, and medico-legal documentation. Edward Angle was the first to incorporate photography into orthodontic diagnosis. Over time, standardized methods, such as those introduced by Goodlin in 1979, have improved the consistency and quality of orthodontic records [1]. Additionally, previous research has demonstrated the role of clinical photography in characterizing smiles and assessing aesthetic parameters, highlighting its importance in dental and orthodontic practice [2].

Previous studies have investigated the use of dental photography in general and pediatric dentistry. For example, Sharland et al. highlighted its applications in patient education, treatment planning, and medico-legal documentation in general practice [3]. Similarly, Alqadi and O’Connell reviewed its use in pediatric dentistry, highlighting its usefulness despite challenges with patient cooperation [4]. Furthermore, the aforementioned research by Cunha et al. explored the impact of age and gender on smile characterization through photographic analysis, reinforcing the relevance of standardized clinical photography in evaluating aesthetic parameters [2]. Moreover, clinical photography has also been utilized in other dental specialties, such as implant dentistry, where it aids in assessing biological sealing around crowns on dental implants [5]. However, the use of photography specifically in orthodontics remains underexplored. There is a need to assess the current practices, challenges, and training needs of orthodontists to address this knowledge gap.

Çifter et al. also explored the patient perspective on dental photography, identifying key concerns such as discomfort with the equipment and insufficient information about the procedures. These findings underscore the need for patient-centered approaches to increase the acceptability of orthodontic photography protocols [6].

In addition, Sandler et al. evaluated the quality of clinical photographs taken by orthodontists, professional photographers, and orthodontic assistants. The study found that orthodontists tended to produce higher-quality intraoral photographs than assistants, but no significant differences were observed in extraoral photographs. These findings suggest that training and standardization can ensure high-quality results regardless of who is taking the photographs [7].

Despite advances in photography and its increasing use in clinical settings, major organizations such as the AAO (American Association of Orthodontists) [8] and SEDO (Spanish Society of Orthodontics) [9] lack comprehensive guidelines, leaving orthodontists without reliable recommendations. Digital photography has become the gold standard, providing cost-effective and high-quality documentation for diagnosis, treatment planning, progress tracking, and medico-legal purposes. However, in Spain, differences in equipment, practices, and training demonstrate the lack of uniform protocols [7].

Dental photography plays a fundamental role in medico-legal documentation in dental practice. Its utility lies in its ability to objectively and accurately record the clinical condition of the patient before, during, and after treatment, thus protecting the rights of both the patient and the professional. In an increasingly litigious environment, clinical photographs serve as detailed visual evidence in the event of claims or litigation, providing a solid basis for the defense of procedures performed in accordance with established medical standards [10].

This study aims to evaluate the photographic practices of orthodontists in Spain, analyzing equipment preferences, editing habits, legal considerations, and self-assessed skill levels. By identifying gaps, this research aims to inform the development of training and protocols to optimize clinical outcomes.

## 2. Materials and Methods

### 2.1. Study Design and Population

This cross-sectional study was approved by the Ethics Committee for Research with Medicines of the Principality of Asturias (CEImPA2024.241) and conducted according to the Declaration of Helsinki (Supplementary Material File S1).

The required sample size was calculated assuming a population of 3200 Spanish orthodontists (data provided by SEDO), an expected standard deviation of 0.9 (based on a pilot study), a precision of 0.1, and a 95% confidence level. This resulted in a required sample of 287 participants. Considering an estimated 5% potential loss, the final sample size was set at 303 participants.

Data were collected between September and October 2024, and participation was voluntary, with no incentives provided.

- Inclusion criteria consisted of licensed dentists practicing orthodontics in Spain. Orthodontists with any level of training in photography, including those who have completed courses in dental photography.
- Exclusion criteria were practicing outside Spain or lack of orthodontic activity.

### 2.2. Procedure

The survey was developed using Google Forms^®^ (Google LLC, web-based, Mountain View, CA, USA) for its accessibility and mobile compatibility. It consisted of a total of 19 questions, including 2 short-answer questions, 12 multiple-choice questions with a single answer, 3 multiple-response questions, and 2 numeric linear scale questions (Supplementary Material File S2):

- Perceived importance of photography for diagnosis (1 to 10 scale). Participants rated how essential they consider clinical photography for diagnostic purposes, with 1 indicating not important at all and 10 indicating extremely important.
- Photographic equipment used. Respondents reported the type of equipment used in their clinics, including mobile phones, compact cameras, DSLR cameras with standard or macro lenses, and DSLR cameras with macro lenses and ring flashes.
- Types of extraoral and intraoral photographs taken during first visits. Participants specified which standard extraoral views (e.g., frontal with lips at rest, frontal in smile, lateral views) and intraoral views (e.g., frontal, occlusal, lateral, frenulum, tonsils) they routinely capture for patient records.
- Self-assessment of photography skills (1 to 5 scale). Participants rated their own photographic proficiency, with 1 indicating poor skill level and 5 indicating high skill level.
- Interest in improving photography skills. The survey assessed whether respondents were interested in enhancing their photography skills, with a binary yes/no response.
- Preferred training formats. Those interested in improving their skills could choose their preferred learning modality, including online theoretical courses, hybrid courses combining online theory with in-person practice, or fully in-person courses.
- Legal aspects of photographic practices. Respondents indicated how they manage patient consent for photography, including whether they use a specific consent form, include it in the general informed consent, or do not obtain consent.
- Editing and cropping techniques. Participants reported whether they edit or crop their images, and if so, which software they use (e.g., Windows Photos, MacOS Photos, Dolphin, mobile phone editors). Additionally, they were asked about their preferred aspect ratios for cropping frontal intraoral and occlusal photographs.
- To ensure the validity and reliability of the survey, it was reviewed and verified by a committee of five experts in legal dentistry and digital photography. This process aimed to enhance the credibility of the questions and ensure that they were both relevant and accurate. The questionnaire was also piloted among a small group of orthodontists to assess feasibility and comprehension.

The survey link was disseminated via scientific societies and official dental councils. All participants provided informed consent before completing the survey. The consent form is available in the Supplementary Materials of this manuscript.

### 2.3. Statistical Analysis

The data obtained, properly coded, were entered into a Microsoft Excel database (Microsoft Corporation, version 2021, Redmond, Washington, DC, USA) for initial organization. Subsequently, they were analyzed using R statistical software (R Development Core Team, version 4.4.1, Vienna, Austria) [11].

A descriptive analysis was performed, providing both absolute and relative frequency distributions for the qualitative variables. For the quantitative variables, measures of central tendency and dispersion were calculated. The relationships between variables were assessed using Pearson’s Chi-square test or Fisher’s exact test, depending on whether the hypothesis about expected frequencies was met. Statistically significant differences were considered for variables with a *p*-value less than 0.05 (*p* < 0.05).

## 3. Results

### 3.1. Participant and Descriptive Data

A total of 304 valid responses were included in the study, while 51 responses were excluded for not meeting the inclusion criteria. Most respondents were female (77.96%), with a mean age of 37.54 years (SD: 9.08). Most participants were between 27 and 42 years old (62.62%), followed by 43–58 years (26.56%), while those younger than 26 (7.21%) and older than 59 (3.61%) were less represented. The demographic characteristics of the study population are summarized in Table 1.

Regarding professional role, 51.64% identified themselves as exclusive orthodontists, followed by general dentists with orthodontic practices (33.55%), pediatric dentists with orthodontic practices (8.88%), and orthodontic students (5.92%). Educational background showed that 58.55% had undertaken a part-time master’s program, 28.29% had completed a full-time master’s program, and 12.5% had undergone modular training. A small proportion (0.66%) described themselves as self-taught.

Professional experience varied, with 36.51% having practiced orthodontics for less than five years, 25% between five and ten years, 16.45% between ten and fifteen years, 12.5% between fifteen and twenty years, and 9.54% more than twenty years. Membership of orthodontic societies also showed variation: 42.11% were members of the Spanish Society of Orthodontics (SEDO), 5.26% of the American Association of Orthodontists (AAO), and 6.25% of the European Orthodontic Society (EOS), while 51.97% did not belong to any professional society.

### 3.2. Photography Practices

Nearly 49% of respondents enjoyed taking photographs in their spare time outside the dental field.

Intraoral photography was widely used by respondents, particularly during initial consultations. Frontal views were universally taken (100%), followed closely by right and left lateral views, with usage rates of 99.67% and 99.34%, respectively. Upper and lower occlusal views were also common, reported by 98.36% and 98.68% of participants, respectively. In addition, less conventional photographs such as lingual frenulum images (17.43%) and tonsil views (12.5%) were taken by smaller subsets of participants.

Extraoral photography was similarly common, with 98.03% of respondents taking frontal smiling views. Resting frontal views and lips closed views were used by 75.33% and 87.17%, respectively. Profile views were taken by 64.47% of respondents when at rest, 85.53% when lips were closed, and 80.26% when smiling. Half-profile shots were less common, taken with lips sealed by 51.64% of respondents, at rest by 40.46%, and while smiling by 59.87%. Zhenital and nadir photos were taken by 21.38% and 18.09% of respondents respectively.

Most respondents (68.09%) relied on DSLR cameras with macro lenses and ring flashes as their primary equipment. Smartphones were the second most popular choice, used by 14.14%, with 5.26% using special dental attachments. Compact cameras (1.64%) and tablets (0.33%) were rarely used.

In terms of editing tools, Windows Photos and MacOS Photos were the most used programs, with 28.95% and 16.12% of respondents, respectively. Other tools included mobile phone editors (9.54%), Keynote (9.54%), and Orthokit (6.25%). Notably, 13.16% of respondents did not crop their images, while 62.83% preferred to crop based on aspect ratio, with 4:3 being the most popular for intraoral (53.29%) and occlusal (84.21%) images.

### 3.3. Legal Aspects of Taking Photographs

Consent practices for photography varied widely. Most respondents (63.82%) included photography in their general consent forms, while 16.45% used a specific consent form for image release. However, 19.74% of participants reported that they did not obtain consent for photography.

### 3.4. Interest in Training

There was a strong interest in training, with 75.99% of respondents expressing a desire to improve their photographic skills. The most preferred training format was a hybrid model combining online theoretical learning with face-to-face practical workshops (38.16%), followed by fully face-to-face courses (35.53%) and fully online courses (26.32%).

### 3.5. Significant Findings

Statistical analysis revealed notable differences in self-reported photography skills.

- Male participants rated their abilities notably higher than female participants (*p* = 0.003).
- Those who practiced photography as a hobby assessed their skill level as superior (*p* > 0.001).
- Participants using DSLR cameras with flash reported greater proficiency compared to those using smartphones (*p* < 0.001). Additionally, they also placed greater importance on photography for diagnosis (*p* = 0.001) and took more photos overall (*p* < 0.001).
- Orthodontists with exclusive practice placed more diagnostic value on photography than general practitioners with orthodontic experience (*p* = 0.019).
- Participants with 5 to 10 years of experience in orthodontics considered photography more important than those with fewer than 5 years of experience (*p* = 0.021).
- Respondents with full-time training assessed their skills more favorably than those with part-time or modular training (*p* = 0.014).
- Those who used a specific consent form for photography took more photos than those who included it in a general consent form (*p* = 0.017) or did not obtain consent at all (*p* = 0.033). Furthermore, they rated their photography skills higher than those who did not sign any consent form (*p* = 0.002) or those who included it in a general consent form (*p* = 0.041). They also attached greater importance to photography (*p* = 0.011 and *p* = 0.034).

## 4. Discussion

The aim of this study was to evaluate the use of dental photography among orthodontists in Spain, focusing on the types of photographs taken, equipment used, self-assessed skills, and legal considerations. The findings revealed considerable variability in photographic practices, with DSLR cameras with macro lenses and ring flashes being the most commonly used equipment, which was also associated with higher self-reported skill levels. While intraoral photographs were almost universally taken, there was greater variation in the use of extraoral images, particularly those with an aesthetic focus. A significant number of participants expressed a desire to improve their skills, with hybrid training formats combining online learning and hands-on workshops being the preferred option. These findings highlight both the essential role of photography in orthodontic diagnostics and the need for standardized protocols to unify practices and improve clinical outcomes.

The predominance of female participants contrasts with previous studies, where male orthodontists were more commonly represented, potentially affecting the generalizability of the findings [12,13]. This shift may affect the generalizability of the findings and warrants consideration.

In our study, where all participants were orthodontists, DSLR cameras (including flash) were the most used equipment, reported by 69.41% of respondents, while smartphones accounted for 19.74%. In contrast, the study by Lazar et al. conducted among general dentists in Romania showed a more balanced distribution, with 51.79% of participants using DSLR cameras and 44.05% relying on smartphones [14]. Compared to our findings, general dentists showed a higher reliance on smartphones, while orthodontists preferred DSLR cameras. As Arya Prasad points out, the continued development of smartphone cameras may eventually allow them to surpass DSLR cameras in photographic quality for intraoral imaging. Although our results suggest that smartphones have not yet had a significant impact on orthodontic practice, this remains a promising area for future research to explore whether technological advances may lead to a shift in this trend over time [15].

Our analysis showed that 63.93% of participants included the use of clinical photographs in a general consent form, compared with 7.6% in a previous study that used written consent without specifying the format, both referring to a similar type of general consent. In addition, 16.39% of participants in our study used a specific written consent form for image use, significantly higher than the 3% observed in the previous study [16]. These findings suggest stricter adherence to legal and ethical standards in orthodontic practice compared to pathology.

Of the participants, 86.88% cropped their images. In comparison, the study by Indu et al. found that 66.7% of postgraduate students and 68.3% of oral pathology faculty used image editing software, with cropping and brightness/contrast adjustment being the most common edits [16]. This suggests that orthodontists are more likely to crop photographs than oral pathologists, possibly due to the importance of obtaining standardized images in orthodontic practice.

In terms of training needs, 76.07% of participants expressed a desire to improve their skills through additional courses. This finding, together with Albugami et al.’s observation that 58.9% of undergraduate students had not received any formal course in photography, emphasizes the need to strengthen photography education during undergraduate studies to meet the apparent demand for better training in this area [17]. In addition, Indu et al. highlighted that the number of participants in their survey who had received no training or exposure to medical photography publications/books was statistically significant (*p* = 0.000), further highlighting the widespread gap in education and awareness in this area [16].

One of the widely used photo-editing programs in this study is OrthoKit, a tool originally designed for photo editing, that has expanded its functionalities to include orthodontic-specific features. These include advanced photo alignment, editing tools, and deep learning techniques for image classification and pattern recognition, aligning with trends in AI-based technology for clinical applications [18,19].

The use of photographs and videos in teledentistry is essential as remote monitoring becomes more common in orthodontics. According to Torres et al., tools such as Dental Monitoring^®^ provide detailed images of the patient’s teeth, allowing effective monitoring of treatment progress without frequent in-person visits [20]. However, for this approach to be successful, orthodontists must master the skills needed to take high-quality photographs and effectively teach patients to do the same. As telemedicine shapes the future of orthodontics, these skills will be critical to ensuring reliable and effective remote treatment.

Certain limitations of this study should be acknowledged. Firstly, the anonymous survey design, while encouraging candid responses, carries a small risk of duplicate submissions, potentially introducing bias. In addition, the voluntary nature of participation may have introduced a selection bias, over-representing orthodontists with a greater interest or skill in photography. This, combined with the exclusive focus on orthodontists in Spain, limits the generalizability of our findings to other countries where photography practices and standards may differ.

Our findings provide a foundation for future research and clinical interventions. Future studies should validate self-reported practices through direct observation or audits to ensure accuracy. Additionally, greater attention should be given to the legal and ethical dimensions of photography, particularly regarding orthodontists’ understanding of and compliance with patient privacy and consent regulations. Expanding the geographical scope of research could offer a broader perspective on global practices, highlighting cross-country differences and similarities.

## 5. Conclusions

This study highlights significant variability in photographic practices among orthodontists in Spain, including differences in equipment, types of photographs taken, and editing preferences. The findings underscore the need for standardized protocols and training programs tailored to modern orthodontic practices. Hybrid training formats combining online theory and face-to-face practice may meet the high demand for improving photographic skills and ensuring consistent diagnostic accuracy.

## Figures and Tables

**Table 1:** Sample Characteristics.

Variable	Category	N (%)
Gender	Female	237 (77.96%)
	Male	67 (22.04%)
Age	<26 years	22 (7.24%)
	27–42 years	190 (62.5%)
	43–58 years	81 (26.64%)
	>59 years	11 (3.62%)
Type of orthodontic practice	Orthodontic student	18 (5.92%)
	General dentists with orthodontic practices	102 (33.55%)
	Pediatric dentists with orthodontic practices	27 (8.88%)
	Exclusive orthodontists	157 (51.64%)
Year of experience	Less than 5 years	111 (36.51%)
	5–10 years	76 (25%)
	10–15 years	50 (16.45%)
	15–20 years	38 (12.5%)
	More than 10 years	29 (9.54%)
Training in Orthodontics	Self-taught	2 (0.66%)
	Modular training	38 (12.5%)
	Part-time master’s program	178 (58.55%)
	Full-time master’s program	86 (28.29%)

## Data Availability

The raw data supporting the conclusions of this article will be made available by the authors on request.

## Supplementary Materials

The following supporting information can be downloaded at: https://www.mdpi.com/article/10.3390/jcm14061984/s1, File S1: Approval from the Ethics Committee for Research with Medicines of the Principality of Asturias; File S2: Questionnaire including informed consent.

## Abbreviations

The following abbreviations are used in this manuscript:

AAO	American Association of Orthodontists
AI	Artificial Intelligence
DSLR	Digital Single-Lens Reflex
SD	Standard Deviation
SEDO	Spanish Society of Orthodontics
EOS	European Orthodontic Society
