# Supplementary material for: Clinical Photography in Orthodontic Practice: Insights from a Nationwide Survey in Spain
Source: J Clin Med. 2025 Mar 14;14(6):1984. doi: 10.3390/jcm14061984 (PMC11943337; doi:10.3390/jcm14061984)
Supplement: Supplementary file 1 [file jcm-14-01984-s001.zip › S1 - Questionnaire including informed consent.pdf]

We have requested your participation in a research study. Before deciding whether to accept participation, it is important that you understand the reasons for conducting the research: how your information will be used, the nature of the study, and the potential benefits, risks, and inconveniences involved.

WHAT ARE THE BACKGROUND AND OBJECTIVE OF THIS STUDY?

There are no previous studies on the use of photography in orthodontics in Spain and Europe. The objective of this study is to quantify and assess the current state of the use of photography for diagnosis in orthodontics.

WHAT ARE THE POSSIBLE SIDE EFFECTS, RISKS, AND INCONVENIENCES ASSOCIATED WITH PARTICIPATION?

The time required to complete the survey.

WHAT ARE THE POSSIBLE BENEFITS OF PARTICIPATING?

Contribution to scientific development in the field of orthodontics.

HOW WILL MY DATA BE USED IN THE STUDY?

The treatment, communication, and transfer of personal data of the participating subjects in the trial comply with the provisions of Organic Law 3/2018, of December 5, on the Protection of Personal Data and guarantee of digital rights.The study has been approved by the Medical Research Ethics Committee of the Principality of Asturias (CEImPA Code 2024.241).

These data do not include your name or address; instead, you will be assigned a response number. The research team will only have access to the assigned number that allows associating the study data with you. However, regulatory authorities, the independent ethics committee, or other supervisory entities may review your personal data. The purpose of such reviews is to ensure the proper conduct of the study or the quality of the study data.

If you withdraw your informed consent to use your data for the study, you will not be able to continue participating in the research. Please note that the study results may be published in the literature, but your identity will not be disclosed.

HOW CAN I ESTABLISH CONTACT IF I NEED TO OBTAIN MORE INFORMATION OR ASSISTANCE?

By accepting consent, you affirm that you have been informed of the study's characteristics,

By accepting consent, you affirm that you have been informed of the study's characteristics, have understood the information, and all your doubts have been clarified.

In case of experiencing any harm related to the study or to obtain answers to any questions that may arise during the research, please contact:

Dr. Brezo Suárez Rodríguez
University of Oviedo
Address: Instituto Asturiano de Odontología C/ Catedrático José Serrano Nº10. 33006
Oviedo (Asturias)

As part of the doctoral thesis being developed at the University of Oviedo, a study * is being conducted on the current state in Spain regarding the use of intraoral and extraoral photography as part of the diagnostic protocol in orthodontics. To gather the necessary information, a survey will be conducted. We invite you to collaborate in this research and contribute your opinions on the study topic through your participation. Although you will not receive any direct benefit from participating, the findings of the study could benefit your community. The information collected may be provided to healthcare professionals and programs working directly or indirectly in the field of orthodontics. According to Organic Law 3/2018, of December 5, on the Protection of Personal Data and guarantee of digital rights, the information you provide for the study will be completely anonymous and confidential. Documents used in interviews will be stored in a secure space accessible only to the study investigator. A code will be assigned to each interview, so your personal data will not be disclosed at any time. The information obtained will only be used to fulfill the purpose mentioned in this letter. The data collected will be analyzed as a group, without the ability to identify study participants. This authorization will remain valid until the end of the study, unless you cancel it earlier. You can cancel this authorization at any time by sending written notice to the Investigator at the following address:
brezo@suarezsolis.com
By accepting this consent, none of the legal rights have been waived.

☐ I agree to participate in the research and authorize the collection of data for scientific and/or dissemination purposes.

Estado actual de la fotografía como método diagnóstico en ortodoncia en España y Europa.

brezosuarez@gmail.com Cambiar de cuenta

No compartido

* Indica que la pregunta es obligatoria

Sección sin título

In which country are you registered as dentist? *

- ◯ Spain
- ◯ Otro: ____________

In which autonomous community's dental college are you registered? *

Tu respuesta ____________

How would you define your professional activity?

- Exclusive orthodontist
- Pediatric dentist with practice in orthopedics and orthodontics
- General dentist with practice in orthodontics
- Orthodontic student
- I do not perform orthodontics

How would you define your orthodontic training? *

- Full-time Master's degree
- Part-time Master's degree
- Modular training
- Self-taught training

How many years of experience do you have in orthodontic practice? *

- Less than 5 years
- 5 - 10 years
- 10 - 15 years
- 15 - 20 years
- More than 20 years

*

How many years of experience do you have in orthodontic practice?

- Less than 5 years
- 5 - 10 years
- 10 - 15 years
- 15 - 20 years
- More than 20 years

Are you a member of any orthodontic association? (Please select all that apply) *

- Yes, of SEDO (Sociedad Española de Ortodoncia y Ortopedia Dentofacial)
- Yes, of AAO (American Association of Orthodontists)
- Yes, of EOS (European Orthodontic Society)
- No
- Otro:

Do you enjoy practicing photography outside of the dental field in your free time? *

- Yes
- No

How important do you consider photography for your diagnosis? *

	0	1	2	3	4	5	6	7	8	9	10	
Insignificant	○	○	○	○	○	○	○	○	○	○	○	Significant (Crucial)

What intraoral photos do you use for diagnosis? (Please select all that apply) *

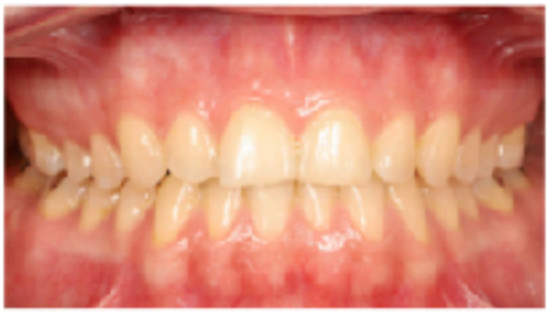

☐ Frontal

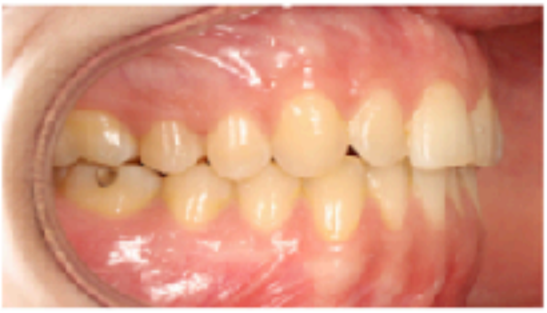

☐ Right lateral

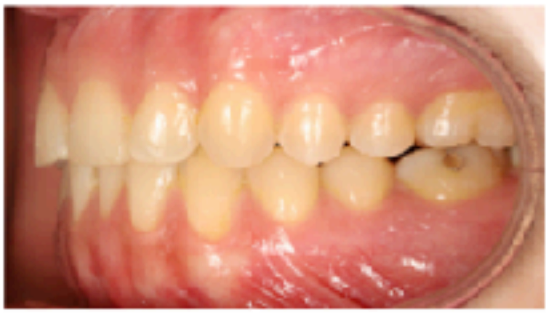

☐ Left lateral

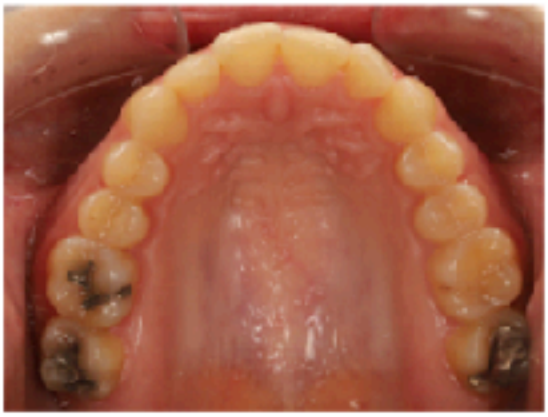

☐ Upper occlusal

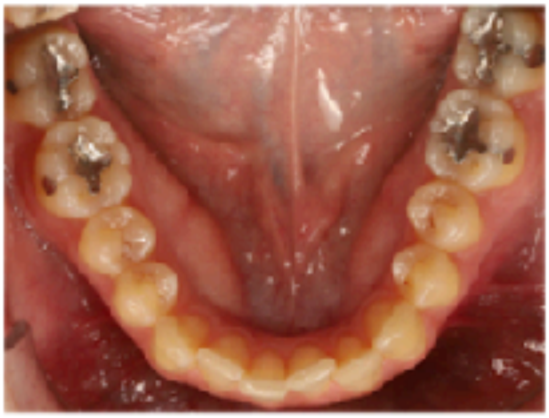

☐ Lower occlusal

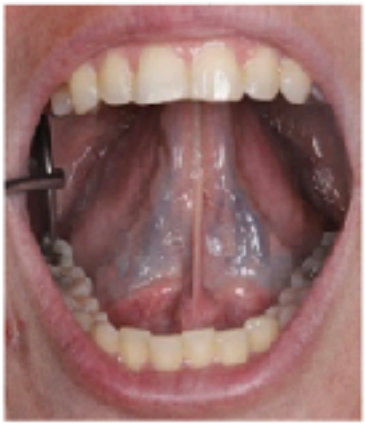

☐ Lingual frenulum

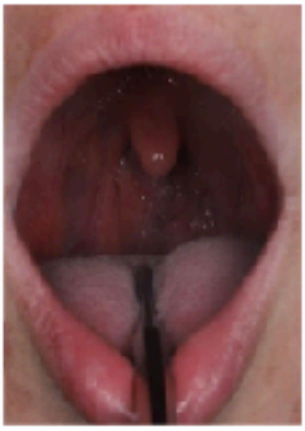

- [ ] Tonsils
- [ ] Otro:

- [ ] I do not take intraoral photographs for diagnosis

What extraoral photos do you use for diagnosis? (Please select all that apply)

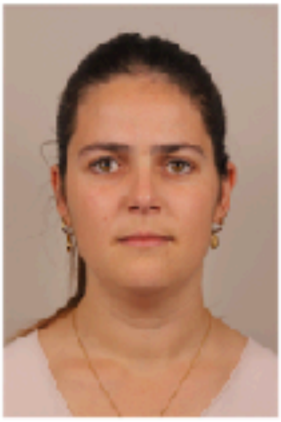

- [ ] Frontal photo with lips sealed

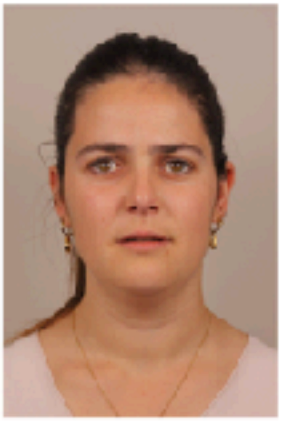

- [ ] Frontal photo with lips at rest

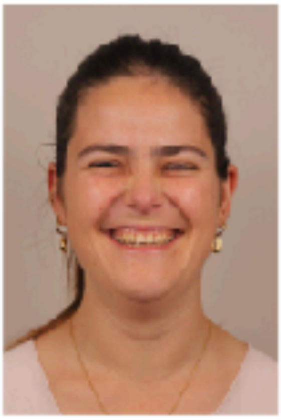

- [ ] Frontal photo smiling

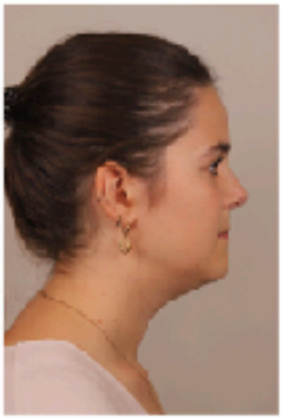

- [ ] Profile photo with lips sealed

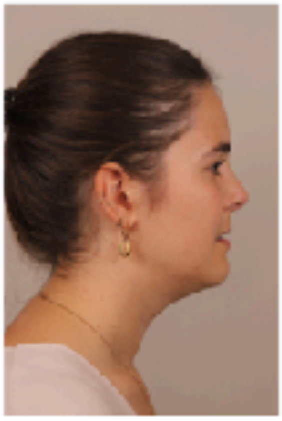

- [ ] Profile photo with lips at rest

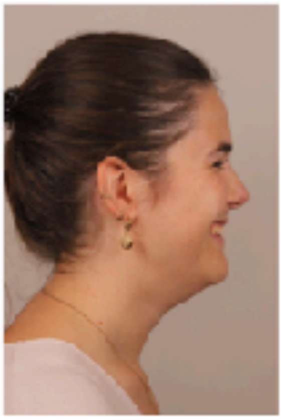

- [ ] Profile photo smiling

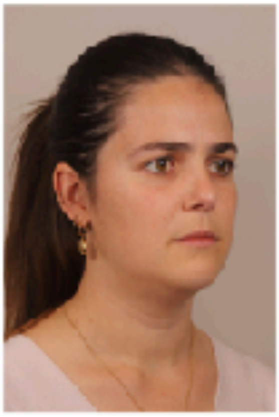

☐ Mid-profile photo with lips sealed

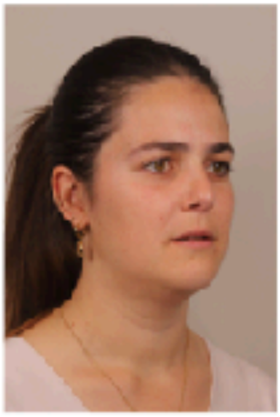

☐ Mid-profile with lips at rest

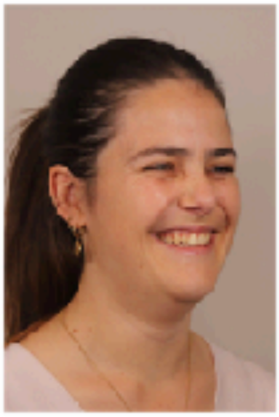

☐ Mid-profile photo smiling

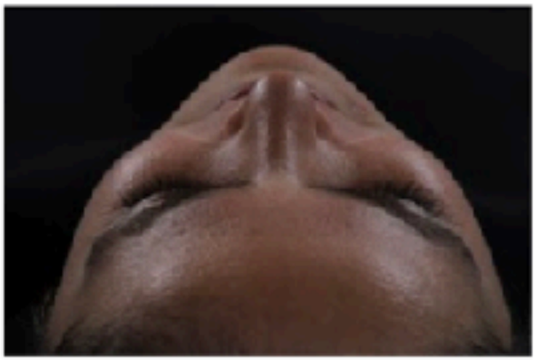

☐ Zenith

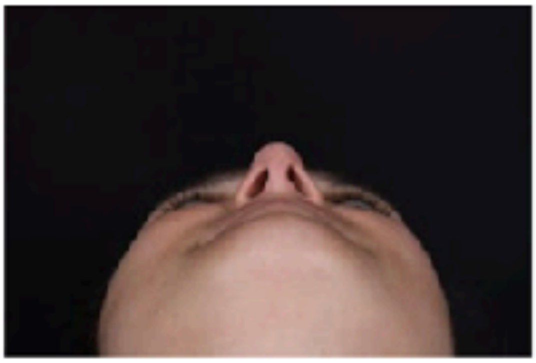

☐ Nadir

☐ I do not take extraoral photographs for diagnosis

Please indicate with which equipment you take photographs in your clinic: *

- ◯ Mobile phone
- ◯ Mobile phone with specific attachment for dental photography
- ◯ Compact camera
- ◯ DSLR camera (Reflex) with standard lens
- ◯ DSLR camera (Reflex) with macro lens
- ◯ DSLR camera (Reflex) with macro lens and ring flash
- ◯ I never take photographs
- ◯ Otro: ____________________

What program do you use to crop and/or edit the photographs you take? *

- ◯ I don't edit or crop photographs
- ◯ Mobile phone editor
- ◯ Orthokit
- ◯ Photos (MacOS program)
- ◯ Photos (Windows program)
- ◯ Patient's PowerPoint
- ◯ Patient's Keynote
- ◯ Dolphin
- ◯ Nemotec

What program do you use to crop and/or edit the photographs you take? *

- I don't edit or crop photographs
- Mobile phone editor
- Orthokit
- Photos (MacOS program)
- Photos (Windows program)
- Patient's PowerPoint
- Patient's Keynote
- Dolphin
- Nemotec
- Custom program
- Otro: ____________

*

How do you usually crop the photographs?

- I don't crop them
- I only crop, without taking into account the aspect ratio (3:2, 16:9...)
- I crop and choose the aspect ratio that seems most appropriate for each photograph (3:2,16:9...)

Which of these cropping options do you find most suitable for intraoral photographs? *

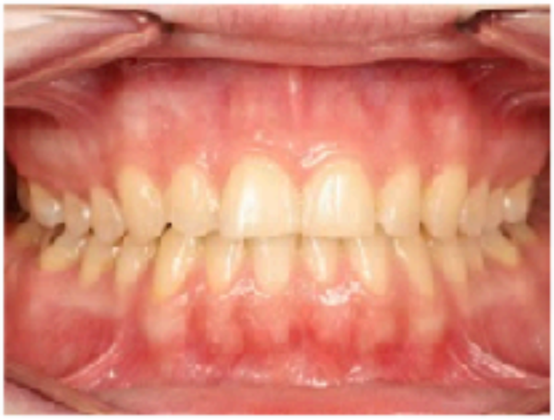

◯ 4/3

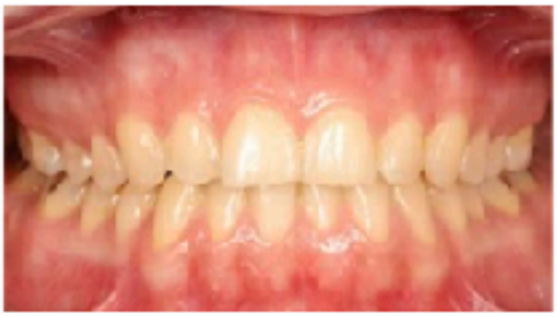

◯ 16/9

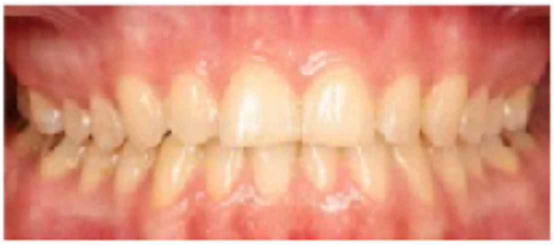

◯ 21/9

Which of these cropping options do you find most suitable for occlusal photographs? *

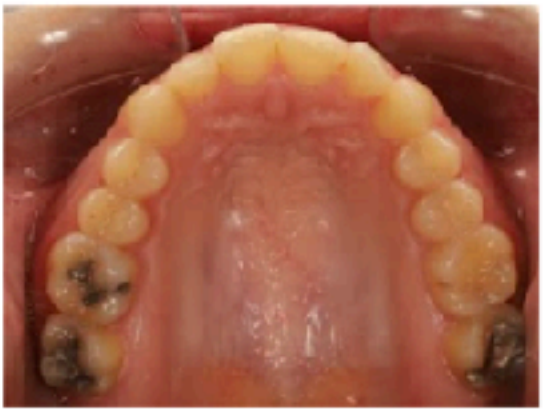

- ○ 4/3

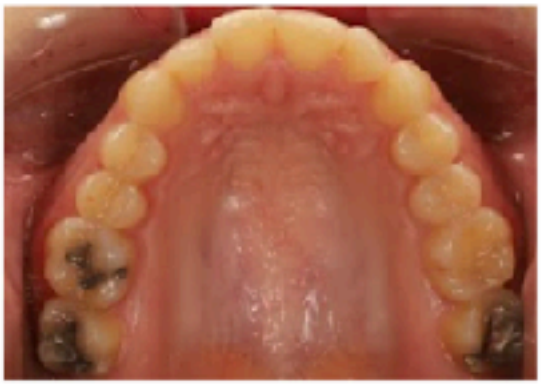

- ○ 10/7

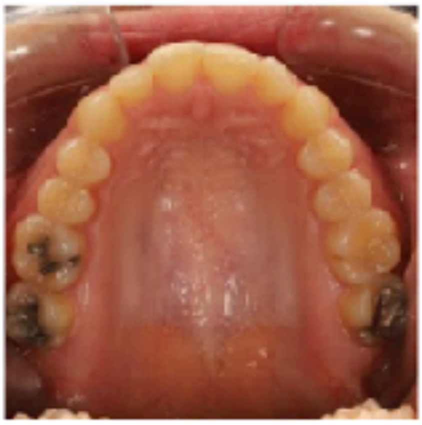

- ○ 1/1

Regarding the legal aspect of taking photographs... *

- ○ I sign a specific image transfer consent
- ○ In the general informed consent, I include the transfer of images

Regarding the legal aspect of taking photographs... *

- ◯ I sign a specific image transfer consent
- ◯ In the general informed consent, I include the transfer of images
- ◯ I do not sign consent

How would you consider your level of photography? *

	1	2	3	4	5	
Very bad	◯	◯	◯	◯	◯	Very good

Would you like to improve your level through courses? *

- ◯ Yes
- ◯ No

What course format would you prefer? *

- ◯ Online theory
- ◯ Online theory and in-person practice
- ◯ In-person theory and practice
