# Supplementary material for: Clinical Photography in Orthodontic Practice: Insights from a Nationwide Survey in Spain
Source: J Clin Med. 2025 Mar 14;14(6):1984. doi: 10.3390/jcm14061984 (PMC11943337; doi:10.3390/jcm14061984)
Supplement: Supplementary file 1 [file jcm-14-01984-s001.zip › S2 - Approval from the Ethics Committee for Research with Medicines of the Principality of Asturias.pdf]

GOBIERNO DEL PRINCIPADO DE ASTURIAS

CONSEJERÍA DE SALUD

Dirección General de Calidad, Transformación y Gestión del Conocimiento

Comité de Ética de la Investigación con Medicamentos del Principado de Asturias
Hospital Universitario Central de Asturias
N-1, S3.19
Avda. de Roma, s/n
33011 Oviedo
Tfno: 9851079 27 (ext. 37927/38028),
ceim.asturias@asturias.org

Oviedo, 19 de septiembre de 2024

El Comité de Ética de la Investigación con Medicamentos del Principado de Asturias, ha revisado el Proyecto (Tesis Doctoral) -cód CEImPA 2024.241, titulado "Estado actual de la fotografía como método diagnóstico en ortodoncia en España y el resto de Europa". Investigador Principal Brezo Suarez Rodriguez, Universidad de Oviedo.

El Comité ha tomado el acuerdo de considerar que el citado proyecto reúne las condiciones éticas necesarias para poder realizarse y en consecuencia emite su autorización.

HIP/CI: Incluido en la encuesta.

Le recuerdo que deberá guardarse la máxima confidencialidad de los datos utilizados en este proyecto.

Fdo: PABLO ISIDRO MARRON
Secretario del Comité de Ética de la Investigación
del Principado de Asturias

CONSEJERÍA DE SANIDAD
COMITÉ DE ÉTICA DE LA INVESTIGACIÓN CON MEDICAMENTOS D[illegible] PRINCIPADO DE ASTURIA[illegible]
